# Estimated mortality rate and leading causes of death among individuals with chikungunya in 2016 and 2017 in Brazil

**DOI:** 10.1590/0037-8682-0580-2019

**Published:** 2020-04-09

**Authors:** Livia Carla Vinhal Frutuoso, André Ricardo Ribas Freitas, Luciano Pamplona de Góes Cavalcanti, Elisabeth Carmen Duarte

**Affiliations:** 1 Ministério da Saúde, Secretaria de Vigilância em Saúde, Brasília, DF, Brasil.; 2 Secretaria Municipal de Saúde de Campinas, Departamento de Vigilância em Saúde, Programa Municipal de Controle de Arboviroses, Campinas, SP, Brasil.; 3 Faculdade de Medicina São Leopoldo Mandic, Campinas, SP, Brasil.; 4 Universidade Federal do Ceará, Departamento de Saúde Comunitária, Fortaleza, CE, Brasil.; 5 Universidade de Brasília, Programa de Pós-Graduação em Medicina Tropical, Brasília, DF, Brasil.

**Keywords:** Chikungunya fever, Mortality, Matched-pair analysis, Death certificate

## Abstract

**INTRODUCTION:**

In 2014, the first cases of autochthonous chikungunya (CHIK) were recorded in Brazil. Lethality associated with this disease is underestimated. Thus, this study aimed to analyze the causes of death among individuals with CHIK in Brazil.

**METHODS:**

A descriptive observational study was conducted on individuals with CHIK who died within 6 months from symptom onset. Data pairing between the Information System for Notifiable Diseases and the Mortality Information System was performed. Deaths were classified according to case confirmation criterion, mention of CHIK in the death certificates (DCs), and disease phase. The lethality rate per 1,000 cases was corrected for underreporting and was estimated according to region, sex, age, years of education, race/color, and cause groups.

**RESULTS:**

We identified 3,135 deaths (mention of CHIK in the DCs, 764 [24.4%]). In 17.6% of these cases, CHIK was the underlying cause. Most deaths occurred in the acute (38.1%) and post-acute (29.6%) phases. The corrected LR (5.7; x1,000) was 6.8 times higher than that obtained from the Information System for Notifiable Diseases (0.8). The highest corrected LRs were estimated for among individuals living in the Northeast region (6.2), men (7.4), those with low years of education and those aged <1 year (8.6), 65-79 years (20.7), and ≥80 years (75.4).

**CONCLUSIONS:**

The LR of CHIK estimates based on information system linkage help to reveal the relevance of this disease as the direct cause or as a cause associated with serious or fatal events, provide timely interventions, and increase the knowledge about this disease.

## INTRODUCTION

Chikungunya (CHIK) is a viral disease caused by the chikungunya virus (CHIKV)^1^_._ It belongs to the genus Alphavirus, which is transmitted mainly by the vectors of the *Aedes* genus*.* This virus was first identified in 1953 in Africa^2^ and later in Asia and Oceania. Meanwhile, the first autochthonous cases in America were only identified in 2013. In Brazil, the first autochthonous cases of CHIK were recorded in 2014 in Oiapoque in the state of Amapá^3^ and in Feira de Santana in the state of Bahia^4^_._

A small proportion of individuals with CHIK are asymptomatic. Meanwhile, in symptomatic cases, the most common manifestations are fever, arthralgia, and exanthema^5^.

Approximately 0.3-1% of symptomatic patients can develop atypical manifestations, with neurological, cardiovascular, pulmonary, hepatic, renal, cutaneous, and ocular involvement, and the atypical manifestations in one-third of the patients are severe and characterized by the need for support for at least one vital function^5^ ^,^ ^6^.

The clinical course of CHIK include the following: the acute phase lasting for up to 21 days, post-acute phase lasting between 22 and 90 days, alternating between periods of temporary improvement and relapse^3^ ^,^ ^7^_,_ and chronic phase, which involves pain in the joints that lasts over 90 days and even years after diagnosis. Studies conducted in some places in Brazil have found chronic cases, with rates varying between 45.7% and 75%^8^_._ Meanwhile, studies in other countries have shown that the rate of chronic cases is approximately 50%^5^ ^,^ ^9^ ^,^ ^10^_._

Deaths can occur in any of these phases, by direct or indirect action of the virus^11^ ^,^ ^12^. The age groups who are at higher risk of severe diseases include neonates who acquire the disease in the perinatal period, elderly^13^ individuals, and those with pre-existing diseases^14^_._

According to the Brazilian Health Ministry data, a total of 552,023 cases were identified between 2014 and 2017 (incidence coefficient of 266 cases per 100,000 inhabitants), 403 deaths were caused by the disease, with a lethality rate (LR) of approximately 0.7 deaths per 1,000 cases^15^ ^,^ ^16^ ^,^ ^17^_._ However, the health care systems are still not alert and sensitive in detecting and recording severe CHIK cases and deaths, thereby resulting in classification errors in death certificates (DCs) and underestimation of the mortality and lethality rate of CHIK. Studies have reported an excess of deaths during the CHIK epidemics in Brazil and in other countries, which may be associated with the disease. However, these cases were not appropriately recorded in the information systems^12^ ^,^ ^18^ ^-^ ^21^_._

Thus, this study aimed to estimate the magnitude and to describe the distribution and declared causes of death among individuals notified to CHIK in Brazil between 2016 and 2017.

## METHODS

This descriptive observational study was based on routine data obtained from the Brazilian Health Surveillance System. 

The study population comprised individuals with CHIK who died within 6 months from symptom onset, regardless of the underlying cause stated in the DC. 

The study population was selected using the following criteria:

- Criterion 1: Individuals notified to CHIK, as reported in the Information System for Notifiable Diseases [Sinan] , who died (according to the Mortality Information System [SIM]) within 6 months after symptom onset, regardless of the underlying cause of death.
- Criterion 2: Individuals whose DCs mentioned CHIK in SIM in the study period even if there was no prior notification of the case in Sinan.

Data for the years 2016 and 2017 in the Sinan and SIM databases were used, and the following procedures were utilized to link these two databases. 

Stage 1: All CHIK cases recorded in Sinan during the study period were identified, regardless of case progression in this system. The database was linked with the complete SIM database for the same period, regardless of the cause of death recorded in this system. Non-deterministic pairing between Sinan and SIM was performed using the following identification variables: name, mother’s name, and date of birth. The similarity (Dice-coefficient) between the two records was assessed by calculating the Bloom Filter created using Python. A detailed description of this method can be found in the study of Schnell^22^. 

Stage 2: The scores obtained from pairing varied between 0 and 10,000. The scores equal to 10,000 were considered as pairs since most cases were from perfect pairs. Meanwhile, scores less than 9,000 were not used. We calculated the time between symptom onset and death, which was obtained using the difference between the date of death (SIM) and date of symptom onset (Sinan). This information was used to exclude pairs for which time to death was greater than 6 months, except when CHIK was mentioned in the DC.

Stage 3: Pairs with scores between 9,000 and <10,000 were considered doubtful pairs and were selected for manual inspection after excluding duplicates. As described previously, the same variables used for linkage were used for final checking and identification of true pairs. 

Stage 4: In cases of death recorded in SIM but were not paired with any CHIK record in Sinan, the DCs mentioning chikungunya (ICD A92.0) as an underlying cause or cause associated with death in the study period were added to the final database for analysis, thereby meeting criterion 2 for the study population.

Stage 5: The last stage involved the exclusion of deaths due to external causes (underlying cause in SIM) and those with final classification as discarded CHIK cases in Sinan, regardless of the information shown in the DC.

Based on the pairing stage, the cases were classified according to the case confirmation criterion (Sinan) and mention of CHIK in the DC (SIM). 

Two categories were considered for the case confirmation criterion: confirmed case (i.e., laboratory-confirmed cases [presence of a specific laboratory test with positive results - real-time polymerase chain reaction [RT-PCR], virus isolation, and IgM or IgG serology) and probable case (i.e., those cases confirmed using the clinical epidemiological criteria or having unknown or blank as their final classification and/or confirmation criterion). 

Regarding the mention of CHIK in the DC, the following categories were analyzed: (1) mention of CHIK as an underlying cause, (2) mention of CHIK but not as an underlying cause, and (3) no mention of CHIK. 

Moreover, the following variables were analyzed: region of residence, sex (male or female), age, years of education, race/color, other underlying and associated causes of death, and phase of the disease in which death occurred. Age was categorized as <1, 1-14, 15-44, 45-64, 65-79, and ≥80 years. Meanwhile, education was categorized according to the years of study, which was as follows: none; 1-3, 4-7, 8-11, and ≥12 years; and unknown. ICD-10 was used to define the underlying causes of death. 

The phase of the disease in which death occurred was based on the time (days) between symptom onset and death, and it was categorized as follows: acute phase, death that occurred within 21 days of symptom onset, post-acute phase, between 22 and 90 days, and chronic phase, more than 90 days from disease onset. The IBM Statistical Package for the Social Sciences software version 22 and Microsoft Excel were used for all analyses. The study was approved by the ethics committee of the University of Brasília Faculty of Medicine Research (report number: 2.523.210).

## RESULTS

**FIGURE 1:**
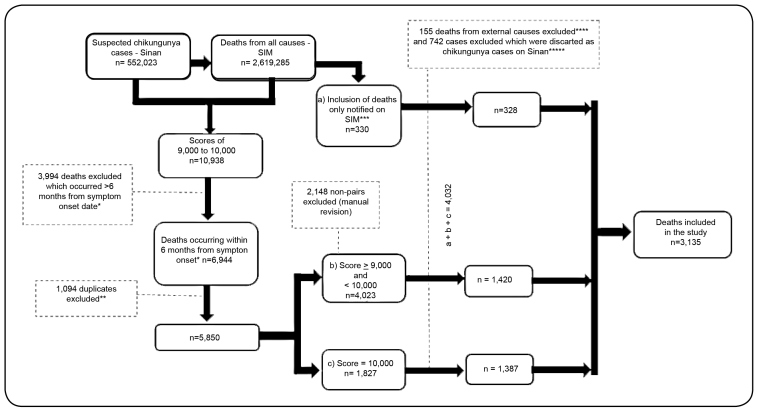
Flow selection of chikungunya patients who died within 6 months in Brazil in 2016 and 2017 using the SINAN and SIM information system linkage process. *Except for individuals with mention of chikungunya in the DC (n=14). **Duplicates generated using the database linkage process when one DC was paired with two cases in SINAN. ***Patients who died with DCs mentioning chikungunya as an underlying or associated cause but was not paired with any cases in SINAN. ****In two of these cases, chikungunya was not an underlying cause. *****In sixteen of these cases chikungunya was mentioning (n=8, underlying cause and n=8, associated cause).

We identified 552,023 suspected cases of CHIK in Sinan in 2016 and 2017 (Figure 1). Of these cases, 10,938 were linked to death notifications (DCs in SIM) in the same period (pairing score of ≥9,000). Based on the eligibility criteria, 3,994 deaths (time to death >6 months) were not included, and 1,094 cases were further excluded owing to duplications generated by pairing, thus, 5,850 cases were finally included.

Of the 5,850 pairs that were included, 1,827 had a score of 10,000 (considered to be perfect pairs), and 1,875 were manually selected from those with a score between 9,000 and <10,000. Furthermore, 330 deaths were added, which had been notified exclusively in SIM with mention of CHIK. Thus, a total of 4,032 deaths were recorded. Finally, 155 deaths due to external causes and 742 deaths considered as discarded CHIK cases in Sinan were excluded. Thus, a total of 3,135 deaths were recorded. 

**FIGURE 2:**
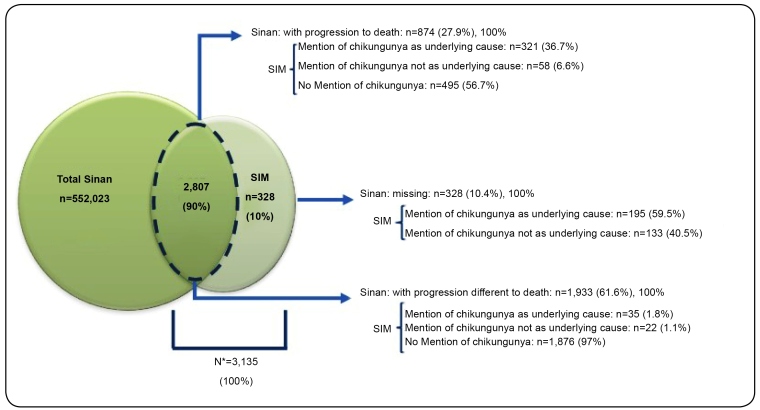
Sources of information about deaths from chikungunya after the SIM and Sinan information system linkage in 2016 and 2017 in Brazil. *All deaths recorded in SIM were included (with or without mention of chikungunya in the DC), which were paired with chikungunya cases recorded in Sinan, plus deaths recorded in SIM with DCs mentioning chikungunya but without pairing with chikungunya cases in Sinan.

Approximately 90% (n=2,807) of deaths had been recorded as deaths in SIM and as cases in Sinan. Meanwhile, 10% (n=328) had been recorded as deaths in SIM but had not been as cases in Sinan (Figures 1 and Figure 2).

Of the 2,807 paired SIM/Sinan notifications, 874 (31%) eventually died based on the data in Sinan (death from CHIK or other causes). Among them, 379 (43.4%) had DCs with mention of CHIK in SIM (Figure 2). In the remaining paired SIM/Sinan notifications (n=1,933, 68.8%), the disease progression field had been filled in with cure or unknown/blank. Among them, 57 (3%) had mention CHIK in SIM. CHIK was identified as the underlying cause of death in 551 (17.6%) of 3,135 patients, and CHIK was mentioned in the DC but was not identified as the underlying cause in 213 (6.8%) patients. Moreover, CHIK was not mentioned in the DCs of 2,371 (75.6%) patients.

Approximately 21.4% of cases had been laboratory-confirmed, and the remainder (78.6%) were classified as probable cases (Table 1). 

CHIK identified as the underlying cause (n=551; 17.6%) was considered a part of the arthropod-borne viral fever and viral hemorrhagic fever groups, which accounted for 20.1% (n=631) of all deaths (Table 1). Among the 551 deaths with CHIK as the underlying cause, 253 were laboratory-confirmed. For both the confirmed and probable cases, arthropod-borne viral and viral hemorrhagic fevers were the most common underlying cause. However, this underlying cause was 2.8 times more common in confirmed cases (40.4%) than in probable cases (14.6%). 

Among the other underlying causes of death, malignant neoplasm (7.9%), influenza and pneumonia (6.9%), ischemic heart disease (6.1%), diabetes mellitus (5.7%), and cerebrovascular diseases (5.5%) are significant (Table 1). No variation was observed in the six leading underlying causes of death for confirmed or probable cases, except for diabetes mellitus, which is more relevant as an underlying cause of death among probable CHIK cases (6.3%). Moreover, conditions occurring in the perinatal period, viral infection of the central nervous system (CNS) and systemic atrophy primarily affecting the CNS, were more commonly observed in confirmed cases (Table 1).

**TABLE 1:** Distribution of underlying causes recorded in SIM for death certificates mentioning chikungunya after SIM/Sinan pairing according to the case confirmation criterion* recorded in Sinan, 2016 and 2017 in Brazil.

Underlying cause group	Confirmed case		Probable case		Total	
	n	%	n	%	n	%
Arthropod-borne and viral hemorrhagic fevers (A90-A99)	271	40.4	360	14.6	631	20.1
Chikungunya (A920)	253	27.4	298	10.8	551	17.6
Malignant neoplasms (C00-C97)	27	4.0	220	8.9	247	7.9
Influenza and pneumonia (J09-J18)	38	5.7	177	7.2	215	6.9
Ischemic heart diseases (I20-I25)	27	4.0	164	6.7	191	6.1
Diabetes mellitus (E10-E140)	26	3.9	154	6.3	180	5.7
Cerebrovascular diseases (I60-I69)	30	4.5	143	5.8	173	5.5
Other types of heart disease (I30-I52)	17	2.5	120	4.9	137	4.4
Ill-defined conditions (R00-R99)	18	2.7	115	4.7	133	4.2
Other bacterial diseases (A30-A49)	13	1.9	81	3.3	94	3.0
Other respiratory system diseases (J60-J99)	15	2.2	79	3.2	94	3.0
Diseases of the blood and certain immune disorders (D50-D89)	21	3.1	70	2.8	91	2.9
Chronic diseases of the lower respiratory tract (J40-J47)	9	1.3	47	1.9	56	1.8
Diseases of the liver (K70-K77)	7	1.0	48	1.9	55	1.8
Kidney failure (N17-N19)	8	1.2	47	1.9	55	1.8
Other urinary system diseases (N30-N39)	9	1.3	45	1.8	54	1.7
Hypertensive diseases (I10-I15)	8	1.2	42	1.7	50	1.6
Systemic atrophy affecting the CNS (G10-G59 and G70-G99)	13	1.9	34	1.4	47	1.5
HIV disease (B20-B24)	4	0.6	41	1.7	45	1.4
Viral infection of the central nervous system (A80-A89)	12	1.8	22	0.9	34	1.1
Certain conditions that occurred in the perinatal period (P00-P96)	5	0.7	5	0.2	10	0.3
Other causes	93	13.9	450	18.3	543	17.3
**Total**	**671**	**21.4**	**2,464**	**78.6**	**3,135**	**100**

Source: Sinan/SIM. *Confirmed case: laboratory-confirmed cases (presence of a specific laboratory test with positive results - real-time polymerase chain reaction, virus isolation, and IgM and IgG serology). Probable case: cases confirmed clinical epidemiological criterion or with the final classification and/or confirmation criterion unknown and blank or deaths identified exclusively in SIM (n=328).

The proportion of deaths (23.9%) that occurred in the chronic phase was higher when CHIK was not mentioned in the DCs. In contrast, the highest proportion of deaths in the acute phase was found when CHIK was mentioned in the DCs (Table 2). 

**TABLE 2:** Phase* of the disease during which death occurred, with mention of chikungunya in the death certificate (DC), and final case classification (confirmed or probable) in 2016 and 2017 in Brazil.

Chikungunya mentioned in the DC	Phase	Confirmed case		Probable case		Total	
		n	%	n	%	n	%
Chikungunya as an underlying cause	Acute	140	57	72	75	212	62
	Post-acute	88	36	19	20	107	31
	Chronic	17	7	5	5	22	6
	**Total**	**245**	**100**	**96**	**100**	**341**	**100**
Chikungunya as the associated cause	Acute	17	43	18	49	35	45
	Post-acute	18	45	10	27	28	36
	Chronic	5	13	9	24	14	18
	**Total**	**40**	**100**	**37**	**100**	**77**	**100**
No mention of chikungunya	Acute	151	41	795	42	946	41
	Post-acute	141	38	653	34	794	35
	Chronic	79	21	466	24	545	24
	**Total**	**371**	**100**	**1.914**	**100**	**2.285**	**100**
**Total**		**656**	**24.3**	**2,047**	**75.7**	**2,703**	**100**

*Cases with inconsistent or missing symptom onset date were excluded from the analysis (n=432).

This study included 3,135 deaths of individuals notified to CHIK. In Sinan, 464 CHIK deaths were recorded in the same period. The corrected lethality rate (CLR; 5.7 per 1,000 cases) was 6.8 times higher than the LR in Sinan alone (0.8 per 1,000 cases). The ratio of these LRs (LR in Sinan) varied according to the groups analyzed. That is, correction had a more significant effect in individuals living in the Northern region (CLR: 13.1 times higher than the uncorrected rate), those aged 15-44 years (7.9 times) and 45-64 years (8.6 times), those who did not attend school (11.8 times), and those with black race/skin color (10.8 times). 

Based on the CLR (per 1,000 cases), the following individuals have a higher risk of death: residents in the Northeast region (6.2); men (7.4); those aged under 1 year (8.6), 65-79 years (20.7), and ≥80 years (75.4); those with low years of education (none: 16.8; 1-3 years: 33.7), and those with white (14.6) or black race/skin color (11.1) (Table 3).

**TABLE 3:** Corrected indicators according to the region of residence and characteristics of patients who died with death certificates mentioning chikungunya in Sinan and/or SIM in 2016 and 2017 in Brazil.

Characteristics	No. of	Incidence	No. of deaths	No. of	Lethality rate	Corrected	Lethality	Relative
	cases*	coefficient	in Sinan	corrected deaths	in Sinan	lethality rate	ratio**	risk***
**Region**								
N	39,466	220.0	9	118	0.2	3.0	13.1	1.6
NE	437,327	763.8	414	2,703	0.9	6.2	6.5	3.3
SE	60,774	69.9	37	273	0.6	4.5	7.4	2.4
S	5,876	19.8	0	11	0.0	1.9	...	1.0
MW	8,872	55.9	4	30	0.5	3.4	7.5	1.8
Unknown/blank	36							
								
**Sex**								
Male	208,249	203.2	237	1,541	1.1	7.4	6.5	1.6
Female	343,671	326.7	227	1,594	0.7	4.6	7.0	1.0
Unknown/blank	431							
								
**Age range (years)**								
<1	7,484	274.2	19	64	2.5	8.6	3.4	5.6
1-14	64,475	149.2	22	106	0.3	1.6	4.8	1.1
15-44	281,567	299.6	54	428	0.2	1.5	7.9	1.0
45-64	139,571	379.7	78	673	0.6	4.8	8.6	3.2
65-79	47,490	425.2	137	982	2.9	20.7	7.2	13.6
≥80	11,695	400.9	154	882	13.3	75.4	5.7	49.6
Unknown/blank	69							
								
**Years of education**								
None	36,782	175.3	52	618	1.4	16.8	11.8	3.4
1-3	23,836	71.6	107	803	4.5	33.7	7.5	6.9
4-7	39,229	117.9	73	486	1.9	12.4	6.7	2.5
8-11	83,725	110.8	90	408	1.1	4.9	4.5	1.0
≥12	19,471	58.8	41	155	2.1	8.0	3.8	1.6
Unknown	349,308		60	495				
								
**Race/skin color**								
White	65,131	71.5	192	949	3.0	14.6	4.9	16.0
Black	16,475	113.5	17	183	1.0	11.1	10.8	12.2
Yellow	4,402	211.2	0	4	0.0	0.9	...	1.0
Brown	301,053	365.9	242	1871	0.8	6.2	7.7	6.8
Indigenous	1,923	235.1	1	7	0.5	3.6	7.0	4.0
Unknown	163,367		12	121				
**Total**	552,351	266	464	3,135	0.8	5.7	6.8	

**Source:** Sinan/SIM and Brazilian Institute of Geography and Statistics - IBGE. *Sinan cases plus 328 cases recovered from SIM. **Corrected Sinan/SIM lethality ratio. ***Corrected lethality rate.

## DISCUSSION

This study first analyzed the causes of death among patients who had CHIK in Brazil based on deaths that occurred within 6 months after diagnosis. This analysis facilitated the identification of a relevant volume of unreported CHIK deaths (85%). 

The proportion of underreporting was relevant in individuals living in the North and Midwest regions, women, those aged 45-64 years, those who were not able to attend school, and those of black race/skin color. The corrected LR might be attributed to CHIK, which indicates a higher risk of death among individuals in the Northeast region, men, those aged <1 year and ≥65 years, those with low years of education, and those of white race/skin color. The causes of death identified alert as to the association of this disease with atypical forms and worsening of pre-existing conditions.

The pairing of the 2016 and 2017 Sinan and SIM databases suggests that the corrected CHIK LR in Brazil (5.7%) in those years might be 6.8 times higher than the LR (0.8%) obtained from Sinan alone. 

Moreover, the data in SIM may have been underreported. Only 24% (764/3,135) of DCs mentioned CHIK as the underlying or associated cause, thereby indicating some limitations in surveillance, healthcare, notification, and DC issuing^23^. Excess deaths (all causes) in SIM were documented during the CHIK epidemic in three Brazilian states, and the estimated number of deaths was 50 times higher than the expected number^21^_._

Factors that can contribute to the underreporting of deaths include access to health services and to conclusive diagnosis and effective case investigation^24^. The unavailability of medical supplies and the recommendation for using the clinical epidemiological confirmation criterion after autochthonous transmission have been assessed^25^ ^,^ ^26^ and are considered barriers in laboratory case confirmation and causal relationship with death. Moreover, CHIK is associated with the decompensation of pre-existing diseases, which are more likely to be considered as the underlying cause of death^24^.

The percentage of chronic CHIK cases may be higher than 45%^8^, and some deaths may occur in this phase^27^. Nonetheless, the Brazilian CHIK surveillance system follows the model established for acute diseases that determines timely case closure within 60 days^26^ ^,^ ^28^. In cases that extend beyond the acute phase, this can have a negative impact on death notification in Sinan.

Based on previous studies, the number of deaths from CHIK was lower than that of deaths from dengue^29^. This phenomenon may have contributed to the low sensitivity in recognizing deaths both in Sinan and SIM. Thus, CHIK was not mentioned in the DCs. However, the re-emergence of CHIK in America has shown that its lethality is high, as observed in Colombia^30^ ^,^ ^31^, Guadeloupe, and Martinique^32^.

Another factor associated with the underreporting of death is the severe atypical forms of the disease^5^ ^,^ ^33^ ^,^ ^34^ and chronification due to the longer interval between symptom onset and death. Moreover, when a lower number of events is recorded in Sinan and SIM, a causal relationship is more challenging to assess. 

The number of deaths in Brazil in 2016 and 2017 might have been higher than that obtained in the current study owing to the lack of diagnosis and underreporting of suspected cases in Sinan, considering that this system was used as the starting point for pairing. A study conducted in Feira de Santana estimated that only 20% of suspected CHIK cases were recorded in 2015^4^. 

Notably, the Zika virus was introduced^36^soon after the detection of the first autochthonous CHIK cases in Brazil in 2014^35^. Thus, some CHIK cases may have been confused with Zika at that time, apart from dengue. Thus, they were not recorded by the surveillance service or were not appropriately classified.

The estimates obtained from the attack rate, the percentage of symptomatic cases, and an estimate of 1 death per 1,000 cases (1/1,000)^37^ in relation to the population of Pernambuco state indicates that there might have been approximately 2,400 CHIK deaths in 2016 in that state alone^23^.

Some authors have found excess deaths among individuals under different age groups in the states of Pernambuco, Bahia, and Rio Grande do Norte in 2015 and 2016. That increase had a strong spatial and temporal relationship with the occurrence of CHIK in those places^21^, indicating that the magnitude found in this study was similar to that of other studies. 

The percentage of patients who died in the acute phase was similar between those who had CHIK as an associated cause and those for whom there was no mention of CHIK in the DCs. Moreover, a considerable percentage of deaths occurred in the chronic phase, as reported in other studies^27^. This result reinforces that even deaths that occurred after the acute phase in this study might have been attributed to CHIK, despite the difficulty of association between the disease and death in the chronic phase^38^.

A higher risk of death was observed in individuals in the Northeast region (6.2%), where transmission was concentrated in 2016 and 2017^17^. 

In this study, the most common characteristics among individuals who died include male sex (7.4%), age under 1 year (8.6%), 65-79 years (20.7%), and 80 years (75.4%), low years of education (none: 16.8%; 1-3 years: 33.7%), and white (14.6%) or black race/skin color (11.1%). 

During the epidemic in Puerto Rico, individuals aged under 1 year and >70 years were 9.2 and 2.4 times, respectively, more at risk of hospital admission than those aged 1-69 years, which is considered the reference age group^40^. Moreover, hospitalization was a factor correlated to disease severity and death. Neonates born to mothers who presented with viremia near childbirth can have severe CHIK with neurological involvement and hemorrhagic manifestations^41^. In Brazil, a study conducted based on surveillance data identified that the median age of death was 75 years^39^. 

In the CHIK seroprevalence studies of adults in Guadeloupe and Martinique in 2014, statistically significant differences were observed between the participants in terms of age and male sex^42^. In contrast, a study conducted in Senegal in 2009 and 2010 has found no difference in terms of sex and age between the confirmed cases of chikungunya^43^. Male sex was significantly associated with the risk of death among individuals in the state of Ceará (OR: 2.05). The same study has found a high proportion of individuals with low education level, and no association was observed between mortality and race/skin color^44^.

Studies have shown that the presence of comorbidities, such as diabetes, hypertension, and kidney disease, were significantly associated with death from CHIK^10^ ^,^ ^39^ ^,^ ^45^, and the number of comorbidities increased with age, which may explain the high number of deaths among individuals aged over 65 years in this study.

Regarding the underlying cause of death identified in the DCs, CHIK was the most common (17.6%), regardless of case classification (confirmed or probable). The other causes include cardiac, liver, and central nervous system diseases^41^. 

Even deaths with underlying causes, such as diabetes, kidney failure, chronic diseases of the upper airways, and hypertensive diseases, may have resulted from the exacerbation of pre-existing diseases, or CHIK may have directly caused these deaths, considering that the decompensation of pre-existing diseases might have increased the risk of death from CHIK^41^.

During the CHIK epidemic in Reunion Island, 610 patients presented with atypical manifestations. Among them, 546 had pre-existing conditions, the most frequent of which are hypertension, diabetes mellitus, cardiovascular diseases, neurological disorders, chronic pulmonary diseases, alcohol abuse, kidney disease, and cancer^6^. Heart disease and hypertension are independently associated with severe disease, and a high number of severe cases and deaths were found to be associated with CHIK^6^.

This study had some limitations that are worthy of attention. The definition of the period between symptom onset and death (6 months) might have indicated that some deaths included in the analysis did not have a direct relationship with CHIK. However, the study considered both death from and death with CHIK. By contrast, defining the 6-month period and the overall study period of 2 years might have indicated that CHIK deaths that occurred outside of this time window were not included. This result raises the hypothesis that the rate of underreporting may be higher. 

The corrected CHIK LR might have been overestimated owing to the possibility of underreported suspected cases, considering that there is a higher risk of reporting severe cases or cases that led to death, rather than benign cases. Moreover, another limitation is that not all cases of CHIK were laboratory-confirmed. Nevertheless, this problem was mitigated by stratifying the analyses as confirmed deaths and probable deaths. 

The other limitations are those inherent to the use of secondary data and probabilistic database linkage. However, these procedures are found in the literature, and all possible procedures were adopted with the aim of minimizing possible pairing and under-recording of errors.

CHIK can cause epidemics with a high number of deaths. In relation this, health professionals must be aware about the importance of early case detection and prevention of death. In particular, they must be alert about atypical forms with high potential for hospitalization and fatalities. 

Surveillance services should aim in detecting CHIK deaths to identify the actual magnitude of the disease, particularly among groups who are at higher risk, and to prevent future deaths^46^. 

The use of Sinan or SIM in calculating the LR of CHIK in Brazil does not identify the magnitude of the problem. The LR of from/with CHIK based on database linkage helped identify the relevance of this disease as a direct cause or as a cause associated with serious or fatal events in the country and provide guidance for timely actions and advancement of knowledge about this disease.

Future studies that use the results of specific tests and clinical data about hospitalizations and deaths can help elucidate and distinguish deaths that are directly or indirectly associated with CHIK.

## References

[1] Schwartz O, Albert ML (2010). Biology and pathogenesis of chikungunya virus. Nat Rev Microbiol.

[2] Lumsden WH (1955). An epidemic of virus disease in Southern Province, Tanganyika Territory, in 1952-53. II. General description and epidemiology. Trans R Soc Trop Med Hyg.

[3] Cunha RV da, Trinta KS (2017). Chikungunya virus: clinical aspects and treatment - A Review. Mem Inst Oswaldo Cruz.

[4] Rodrigues Faria N, Lourenço J, Marques de Cerqueira E, Maia de Lima M, Pybus O, Carlos L (2016). Epidemiology of Chikungunya Virus in Bahia, Brazil, 2014-2015. PLOS Currents Outbreaks.

[5] Thomas Cerny, Schwarz M, Urs Schwarz, Lemant J, Gérardin P, Keller E (2017). The range of neurological complications in chikungunya fever. Neurocrit Care.

[6] Economopoulou A, Dominguez M, Helynck B, Sissoko D, Wichmann O, Quenel P (2009). Atypical Chikungunya virus infections: clinical manifestations, mortality and risk factors for severe disease during the outbreak on Reúnion. Epidemiol Infect.

[7] Simona F, Javelle E, Cabie A, Bouquillard E, Troisgros O, Gentile G (2015). French guidelines for the management of chikungunya (acute and persistent presentations). Med Mal Infect.

[8] Dias JP, Costa MDCN, Campos GS, Paixão ES, Natividade MS, Barreto FR (2018). Seroprevalence of Chikungunya Virus after Its Emergence in Brazil. Emerg Infect Dis.

[9] Burt FJ, Chen W, Miner JJ, Lenschow DJ, Merits A, Schnettler E (2017). Chikungunya virus: an update on the biology and pathogenesis of this emerging pathogen. Lancet Infect Dis.

[10] Cavalvanti LPG, D’angelo SM, Lemos DRQ, Barreto FKA, Siqueira AM, Miyajima F (2018). Is the recent increment in attributable deaths to type-2 diabetes (T2D) associated with the latest chikungunya outbreak in a major epidemic area in Brazil?. Rev Soc Bras Med Trop.

[11] Pialoux G, Gaüzère B-A, Jauréguiberry S, Strobel M (2007). Review Chikungunya, an epidemic arbovirosis. Lancet Infect Dis.

[12] Josseran L, Paquet C, Zehgnoun A, Caillere N, Tertre AL, Solet J-L (2006). Chikungunya Disease Outbreak, Reunion Island. Emerg Infect Dis.

[13] Caglioti C, Lalle E, Castilletti C, Carletti F, Capobianchi MR, Bordi L (2013). Chikungunya virus infection: an overview. New Microbiol.

[14] Badawi A, Ryoo SG, Vasileva D, Yaghoubi S (2018). Prevalence of chronic comorbidities in chikungunya: A systematic review and meta-analysis. Int J Infect Dis.

[15] Ministério da Saúde (MS). Secretaria de Vigilância em Saúde (2016). Monitoramento dos casos de dengue, febre de chikungunya e febre pelo vírus Zika até a Semana Epidemiológica 52, 2015.

[16] Ministério da Saúde (MS). Secretaria de Vigilância em Saúde (2017). Monitoramento dos casos de dengue, febre de chikungunya e febre pelo vírus Zika até a Semana Epidemiológica 52, 2016.

[17] Ministério da Saúde (MS). Secretaria de Vigilância em Saúde (2018). Monitoramento dos casos de dengue, febre de chikungunya e febre pelo vírus Zika até a Semana Epidemiológica 52, 2017.

[18] Freitas ARR, Alarcón-Elbal PM, Paulino-Ramírez R, Donalisio MR (2018). Excess mortality profile during the Asian genotype chikungunya epidemic in the Dominican Republic, 2014. Trans R Soc Trop Med Hyg.

[19] Brito CAA, Teixeira MG (2017). Increased number of deaths during a chikungunya epidemic in Pernambuco, Brazil. Mem Inst Oswaldo Cruz.

[20] Freitas ARR, Gérardin P, Kassar L, Donalisio MR (2019). Excess deaths associated with the 2014 chikungunya epidemic in Jamaica. Pathog Glob Health.

[21] Freitas ARR, Cavalcanti LPG, Von Zuben AP, Donalisio MR (2017). Excess Mortality Related to Chikungunya Epidemics in the Context of Co-circulation of Other Arboviruses in Brazil. PLoS Curr.

[22] Schnell R., Bachteler T, Reiher J (2009). Privacy-preserving record linkage using Bloom filters. BMC Med Inform Decis Mak.

[23] Brito CAA (2017). Alert: Severe cases and deaths associated with Chikungunya in Brazil. Rev Soc Bras Med Trop.

[24] Cavalcanti LPG, Escóssia KNF, Simião AR, Linhares PMC, Lima AAB, Lopes KW (2019). Experience of the Arbovirus Death Investigation Committee in Ceará, Brazil, in 2017: advances and challenges. Epidemiol Serv Saude.

[25] Ministério da Saúde (MS). Secretaria de Vigilância de Saúde (2015). Monitoramento dos casos de dengue e febre de chikungunya até a Semana Epidemiológica (SE) 53 de 2014.

[26] Ministério da Saúde (MS). Secretaria de Vigilância em Saúde (2014). Febre de Chikungunya. Guia de Vigilância em Saúde.

[27] Lima AS, Sousa GS, Nascimento OJ, Castroid MC (2019). Chikungunya-attributable deaths: A neglected outcome of a neglected disease. PLoS Negl Trop Dis.

[28] Ministério da Saúde (MS). Secretaria de Vigilância em Saúde (2006). Sistema de Informação de Agravos de Notificação - Sinan: normas e rotinas.

[29] Ministério da Saúde (MS). Secretaria de Vigilância em Saúde, Secretaria de Atenção Básica (2015). Febre de chikungunya: manejo clínico.

[30] Cardona-Ospina JA, Paniz-Mondolf AE, Rodríguez-Morales AJ (2015). Mortality and fatality due to Chikungunya virus infection in Colombia. J Clin Virol.

[31] Pan AmericanHealth Orgnization - PAHO, World Health Organization - WHO (2015). Number of Reported Cases of Chikungunya Fever in the Americas, by Country or Territory 2015 (to week noted).

[32] Pan American Health Organization (PAHO)/ World Health Organization (WHO) (2014). Number of Reported Cases of Chikungunya Fever in the Americas, by Country or Territory 2013-2014 (to week noted).

[33] Crosby L, Perreau C, Madeux B, Cossic J, Armand C, Herrmann-Storke C (2016). Severe manifestations of chikungunya virus in critically ill patients during the 2013-2014 Caribbean outbreak. Int J Infect Dis.

[34] Alvarez MF, Bolivar-Mejía A, Rodriguez-Morales AJ, Ramirez-Vallejo E (2017). Cardiovascular involvement and manifestations of systemic Chikungunya virus infection: A systematic review. F1000 Fac Rev.

[35] Teixeira MG, Andrade A, Costa MN, Castro J, Oliveira F, Goes CSB (2015). East/Central/South African Genotype Chikungunya Virus, Brazil, 2014. Emerg. Infect. Dis.

[36] Campos GS, Bandeira AC, Sardi SI (2015). Zika Virus Outbreak, Bahia, Brazil. Emerg Infect Dis.

[37] Mavalankar D, Shastri P, Raman P (2007). Chikungunya epidemic in India: a major public-health disaster. Lancet Infect Dis.

[38] Figueiredo LTM (2017). Large outbreaks of chikungunya virus in Brazil reveal uncommon clinical features and fatalities. Rev Soc Bras Med Trop.

[39] Simião AR, Barreto FKA, Oliveira RMAB, Cavalcante JW, Lima AS, Barbosa RB (2019). A major chikungunya epidemic with high mortality in northeastern Brazil. Rev Soc Bras Med Trop.

[40] Hsu CH, Cruz-Lopez F, Torres DV, Perez- Padilla J, Lorenzi OD, Rivera A (2019). Risk factors for hospitalization of patients with chikungunya virus infection at sentinel hospitals in Puerto Rico. Plos Neglected Trop Dis.

[41] Rajapakse S, Rodrigo C, Rajapakse A (2010). Atypical manifestations of chikungunya infection. Trans R Soc Trop Med Hyg.

[42] Gallian P, Leparc-Goffart I, Richard P, Maire F, Flusin O, Djoudi R, Chiaroni J (2017). Epidemiology of Chikungunya Virus Outbreaks in Guadeloupe and Martinique, 2014: An Observational Study in Volunteer Blood Donors. Plos Neglected Trop Dis.

[43] Sow A, Faye O, Diallo M, Diallo D, Chen R, Faye O (2009). Chikungunya Outbreak in Kedougou, Southeastern Senegal in 2009-2010. Open Forum Infect Dis.

[44] Silva GBD, Pinto JR, Mota RMS, Pires RDJ, Daher EF (2019). Risk factors for death among patients with Chikungunya virus infection during the outbreak in northeast Brazil, 2016-2017. Trans R Soc Trop Med Hyg.

[45] Barreto FKA, Montenegro RM, Fernandes VO, Oliveira R, Batista LAA, Hussain A (2018). Chikungunya and diabetes, what do we know?. Diabetol Metab Syndr.

[46] Cavalcanti LPG, Freitas ARR, Brasil P, Cunha RV (2017). Surveillance of deaths caused by arboviruses in Brazil: from dengue to chikungunya. Mem Inst Oswaldo Cruz.

